# How Does Contraceptive Use Affect Women’s Sexuality? A Novel Look at Sexual Acceptability

**DOI:** 10.3390/jcm11030810

**Published:** 2022-02-03

**Authors:** Salvatore Caruso, Gaia Palermo, Giuseppe Caruso, Agnese Maria Chiara Rapisarda

**Affiliations:** Research Group for Sexology, Department of General Surgery and Medical Surgical Specialties, Gynecological Clinic, University of Catania, Via Santa Sofia, 78, 95125 Catania, Italy; gaia.palermo.17@gmail.com (G.P.); giu.caruso97@gmail.com (G.C.); rapisardaagnesemc@gmail.com (A.M.C.R.)

**Keywords:** hormonal contraception, long-acting reversible contraceptive, quality of life, sexual arousal and desire, sexual behavior, short-acting reversible contraceptive

## Abstract

Among the components of a healthy life, sexuality is essential, contributing to both the psychophysical and social well-being of women and, consequently, to their quality of life. A poorly investigated standpoint is the acceptability of contraceptive methods, both in terms of their tolerability and metabolic neutrality and in terms of their impact on sexual life. In this context, we will provide an overview of the different methods of contraception and their effects on female sexuality, from biological changes to organic, social, and psychological factors, which can all shape sexuality. A MEDLINE/PubMed review of the literature between 2010 and 2021 was conducted using the following key words and phrases: hormonal contraception, contraceptives, female sexual function, libido, sexual arousal and desire, and sexual pain. Recent studies have supported the effects of contraceptives on women’s sexuality, describing a variety of positive and negative events in several domains of sexual function (desire, arousal, orgasm, pain, enjoyment). However, satisfaction with sexual activity depends on factors that extend beyond sexual functioning alone. A more holistic approach is needed to better understand the multitude of factors linked to women’s sexuality and contraception. Contraceptive counseling must consider these important elements since they are closely related to good compliance and maximize non-contraceptive health benefits.

## 1. Introduction

Reproductive and sexual health represent a human right that has to be defended and preserved. The aim of contraception is to avoid undesired pregnancies and assure a satisfying sexual life free from procreative risks.

The successful control of fertility steers a woman toward great benefits from personal, economic, and cultural autonomy to psychological and physical welfare [1].

A holistic approach to contraceptive requirements has to consider the individual’s reproductive and sexual health needs. Physicians should use simple, understandable language when counseling about potential risks, benefits, and uncertainties to enable women to choose the best contraceptive for themselves. Counseling is mainly aimed at supporting women’s choices. Different variables can affect the choice of contraceptive and include the woman’s subjective characteristics, as well as the ready availability and straightforward usage of the method [2].

The main goal of modern contraception has been to allow the woman and her partner to live the sexual experience free from the worry of an unwanted pregnancy. Although these are good intentions, at least at the beginning of the use of hormonal contraception, possible interference with the quality of sexual life, desire, arousal, and the level of acceptability in women using hormonal contraceptives has not been well investigated. In the past, few investigations have reported whether hormonal contraception can positively or negatively affect the sexual function of users in different domains of female sexuality, such as desire, arousal, orgasm, lubrication, enjoyment, and pain [3].

However, it is important to emphasize that many factors beyond sexual function itself influence satisfaction with sexual activity. In fact, if sociocultural variables can modulate female sexuality in its qualitative and quantitative aspects, on the one hand, it is also true that the hormonal changes promoted by hormonal contraception could influence sexual habits [3]. Physiologically, in women who do not use hormonal contraceptives or who have taken non-hormonal contraceptives, sexual desire increases during the periovulatory phase of the menstrual cycle [4]. At midcycle, the gradual increase in estrogen promotes the thinning of the cervical mucus, which results in watery secretion with low viscoelasticity. Moreover, androgens are required for the synthesis of the glycoproteins needed for mucous formation [5]; this may explain the decreased vaginal lubrication noted by some women when they are using a hormonal contraceptive with antiandrogenic activity. In addition, hypoandrogenism may cause the onset of vulvodynia and contribute to impaired vaginal lubrication [6,7]. On the other hand, the increase in progesterone during the luteal phase promotes mucus reduction, with less water and a barrier to sperm cells [8]. The increase in cervical mucus and vaginal lubrication may activate the phase of genital arousal, which could b capable of centrally turning on sexual interest [9].

Over the past decade, an increasing number of studies have been engaged in investigating the influences of contraception on sexuality and, above all, the association between hormonal contraception and libido [10].

Even so, a global approach is necessary to study the many facets of female sexuality and the influence of contraception on users. Investigating these issues with greater interest than what has been shown up to now could bring benefits to female sexual well-being and foster a wider use of hormonal contraception.

In this review, we will focus on the different methods of contraception based on their steroid compounds, the route of administration, and regimen, as well as their impact on female sexuality, extrapolated from the most recent literature articles.

## 2. Materials and Methods

We conducted a comprehensive search of the literature using MEDLINE and PubMed. The research covered the period from 2011 to 2021. We used combinations of the following search terms: female sexual function, hormonal contraception, libido, long-acting reversible contraceptive, quality of life and short-acting reversible contraceptive, sexual arousal and desire disorders, sexual pain, and sexual behavior. Titles and abstracts were reviewed by the authors to assess their relevance to the review. Eligibility criteria also included a comparison between two or more contraceptive methods and their influence on female sexual health and the change in users’ quality of life. The following criteria were used to exclude articles: unpublished reports, unspecified date, and the site of the study or suspicion of duplicate reporting. Efforts were made to ensure that there was no overlap within the results and that no case was counted twice. Variables extracted and analyzed included an improvement or deterioration in sexual health—mainly of desire, arousal, lubrication, and sexual pain.

**Figure A1:**
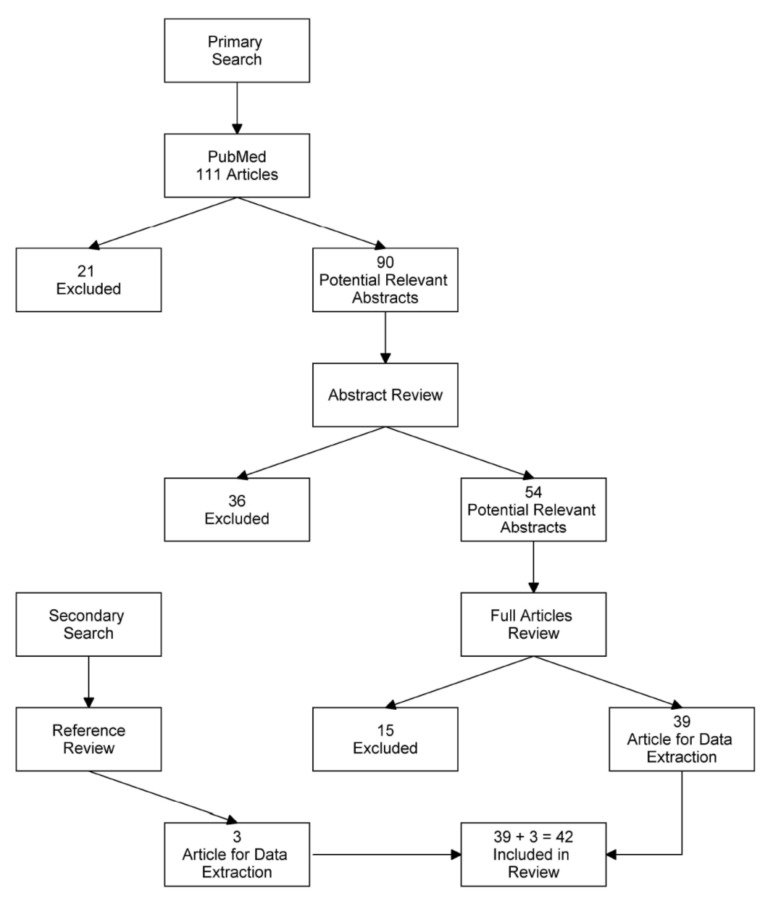
PRISMA flow diagram.

This systematic review had no “stand-alone” study protocol. For reporting outcomes for the ongoing study, the PRISMA guidelines were adopted [Appendix A, Figure A1].

## 3. Results

We analyzed more than one hundred abstracts; however, the quality of sexual life, desire, arousal, and the level of acceptability of hormonal contraception in women were not well investigated.

### 3.1. Combined Oral Contraception and Sexuality

Over the last fifty years, many studies have been drawn up on cultural and social aspects related to hormonal contraceptives [11].

Firstly, the impact of “the pill” on society has been misunderstood. Despite all the social and moral controversies and the scientific debate on side effects, hormonal contraception has, nowadays, obtained a consolidated role [12]. Combined oral contraception (COC) is a reasonable and reversible method of contraception, available with a wide modality of administration and a wide choice of doses of administration, formulations, and regimens.

COC could have advantages in female sexuality, including increasing intra-partner satisfaction and the number of instances of sexual intercourse; resolving painful gynecological symptoms, such as those due to dysmenorrhea or endometriosis, or bothersome symptoms, such as menorrhagia and alterations in the menstrual cycle; and improving the signs and symptoms of clinical hyperandrogenisms, such as acne and hirsutism [13]. However, it could have disadvantages in relation to sexual desire, arousal, lubrication, pain, and orgasm. In fact, sexual side effects are considered to be among the factors promoting contraceptive discontinuation or switching [14].

Hypoactive sexual desire disorder is the most frequently reported sexual symptom among women using hormonal contraception. In a study of 3740 women, the authors observed that 43% of them had experienced a reduction in sexual desire attributed to the use of hormonal contraceptives, compared with 12% of women who used hormone-free contraceptives [15].

To support these data, there is also an Egyptian study on 8422 women using progestin-only contraceptives, in which a significant general worsening in sexuality in terms of desire, arousal, lubrication, and orgasm was reported.

Smaller concentrations of estrogens and progestogen with antiandrogenic activity can lead to vulvo-vaginal atrophy due to reduced activation of sex steroid receptors in the case of a relative lack of estrogen; conversely, the effects of reduced androgen concentrations have not yet been elucidated [16]. On the other hand, current hormonal contraception is characterized by a frankly antiandrogenic pharmacological activity due to the characteristics of both estrogen and progestin [17].

According to recent studies, the dose of ethinylestradiol (EE) can affect the blood levels of free testosterone (FT), reducing it below a critical threshold on the basis of its dosage, and potentially leading to at least one group of women (those most sensitive to steroid variations) suffering from hypoactive sexual desire [18]. Its activity is coupled with the anti-androgenic activity of progestogen [19].

Just over 10 years ago, COCs containing natural estrogen, namely estradiol valerate (E2V) and 17β-estradiol (17β-E), were marketed. E2V and (17β-E) have a lower impact than EE on the synthesis of hepatic proteins, such as the sex hormone-binding globulin (SHBG) [20,21,22].

The assumption of a detrimental effect of the antiandrogenic progestogen on female sexual function was challenged by the STABLE study, a multicenter, randomized, double-blind study on either E2V/dienogest (DNG) or EE/levonorgestrel (LNG). Both treatments were associated with a significant increase in the sexual function of the users between baseline and end-of-study, and resulted in similar improvements in desire, arousal, satisfaction, orgasm, and lubrication [23]. The authors supported the non-difference in sexual function to be due to the progestin component, dienogest with antiandrogenic activity, and levonorgestrel with androgenic activity. They deduced that both formulations had the same effects on sexual function. They did not consider the effects of the two estrogens, E2V and EE, on the modulation of plasma SHBG levels. E2V has a lower estrogenic effect than EE. Therefore, it can be hypothesized that the increase in SHBG is more reduced in the contraceptives with E2V than in those with EE. Hence, hypoandrogenism appears less marked despite the antiandrogenic properties of DNG [20,22].

These results are consistent with other studies investigating the effect of E2V/DNG on the quality of sex life of women who requested a contraceptive to avoid pregnancy [24]. Finally, hypoandrogenism induced by E2V/DNG is milder than that of other COCs containing EE combined with progestogen with antiandrogenic activity [21]. This could be caused by two mechanisms as follows: EE can raise the level of the SHBG and consequent decrease in FT; however, it can directly suppress the ovary androgen production [25]. Moreover, continuous or extended regimens of administration have been associated with supplementary positive variation in a multitude of sexual acceptability factors, from sexual function and libido to a reduction in dysmenorrhea, duration of withdrawal bleeds, and breast tenderness [18]. It is also true that most of the users of hormonal contraceptives do not undergo a reduction in libido even though, in most studies, a decline in plasma levels of FT and an increase in SHBG were demonstrated [26]. Pastor et al., showed that libido decreased only in women using oral contraceptives that contained 15 µg of EE but not in those using COCs containing 20 or 35 µg of EE. Nonetheless, the reduction in vaginal lubrication with sexual arousal disorder reported by some users could be due to the low peripheral dose of EE [27]. Experiencing reduced desire seems to be a strong predictor for changing or suspending a contraceptive method [28]. 

On the other hand, several studies correlate the use of COCs with changes in sexual function—particularly sexual desire and arousal, frequency of sexual activity, and orgasm achievement—but not with enjoyment of sexual activity [28,29].

However, it is still unclear whether these changes in sexuality are directly related to hormonal effects or pill-induced mood swings, or are primarily psychological rejection reactions to fertility control [26].

Another factor that can positively influence women’s sexuality is the certainty of not wanting children. Hormonal contraceptives are a highly effective form of contraception; they help to eliminate anxiety related to the fear of pregnancy, encouraging an easier and more enjoyable sexual experience. Differences in terms of anti-androgenic results and influence on sexuality could be attributed, in part, to the known effects of estrogen on SHBG synthesis and, in part, to the androgenic or anti-androgenic activity of the involved progestin [30]. In conclusion, every woman has her own sensitivity to sexual steroids; therefore, it is not possible to simplify the contraceptive choice. Thus, an effort is strongly required to adopt a hormonal contraceptive that is adapted to her subjective needs. It is essential that counseling considers the woman’s expectations concerning the effects of hormonal contraception on her own sexual activity. In fact, most women expect an improvement in sexuality while they are on hormonal contraception; a worsening or lack of change could lead to discontinuation [31].

### 3.2. Vaginal Ring and Sexuality

The contraceptive vaginal ring (CVR) is an appropriate method for women with indications for hormonal contraception (HC) who have previously experienced sexual dysfunction using oral contraceptives [32].

Many studies that compare COCs and CVRs report that there is no difference in terms of efficacy to inhibit ovulation. The only difference is the compliance of women, maybe also thanks to the elimination of daily intake [33].

Some authors have shown that CVR, in addition to contraceptive efficacy, could promote a positive effect on the couple’s sexuality as a whole, and amplify the complicity and satisfaction of both the partners [34].

Furthermore, in previous studies, women using CVR had better outcomes in relation to desire and sexual satisfaction compared with women using oral hormonal contraceptives. In fact, CVR users had higher sexual desire than those using oral contraceptives combined with EE or desogestrel-only pills [35].

In addition, other authors evaluated sexual efficacy and function by a prospective study involving a prolonged regimen of CVR. An improvement in sexual function and a reduction in sexual discomfort were observed after two months of use [36].

Unlike these, other investigations obtained conflicting results. Indeed, decreased sexual desire was observed more frequently in women using CVR than in those who were intaking oral contraceptives containing 30 µg of EE and 3 mg of DRSP [37]. Today, new polymer compositions are produced for use in the manufacture of CVRs. Their characteristic is to give greater stability and a better withdrawal of steroids from the CVR. In addition to having reduced adverse events, such as spotting, the users report a better QoL and better sexual function when interviewed [38].

In favor of these data, there are many comparisons between the vaginal ring, other types of contraception, and no hormonal contraception. Some authors concluded, from the results, that women who use HC through any route of administration experience an improvement in their sexuality, evidenced by positive variations of sexual interest and fantasies and orgasm intensity and satisfaction; they supported such an improvement by relating it to the reduction in their anxiety and discomfort during unprotected sex of women before starting HC [23,39].

All studies cited relate to the once-a-month ring through which 15 mg of EE and 120 mg of etonogestrel are released daily. 

### 3.3. Progestin-Only Pill (POP) and Sexuality

The most common contraceptive used in Europe during breastfeeding is a POP that contains low doses of desogestrel. Clinical trials of POPs have proved no effects on breastfeeding performance and, consequently, no harmful events in breastfed newborns [39]. Authors noted in a double-blind, placebo-controlled study that POP users have no adverse effects on sexual function compared to women using HC. Overall, data provide reassurance that POPs are unlikely to have a major impact on sexual desire [40]. However, some authors have postulated that progestogen POPs with antiandrogenic activity may adversely affect sexual interest and fantasies. The findings of the studies reinforce the hypothesis that that the effect on sexual function depends on the particular type of progestin and not its dosage [41]. Reports correlating a levonorgestrel-containing combined contraceptive with a desogestrel-containing one have shown different impacts on SHBG concentrations [42]. Moreover, it has been proposed that desogestrel, for its androgenic activity, may exert positive effects on libido [43]. The question of how a different dose or type of progestin could contrastingly affect female sexuality is worthy of additional research.

### 3.4. Intrauterine Devices and Sexuality

The high contraceptive efficacy of long-acting reversible contraceptives (LARC) is the most important characteristic of this kind of intrauterine device. For this reason, they are widely used in current programs and policies of family planning [44]. Nowadays, women who use SARCs, such as oral, patch, or vaginal combined hormonal contraceptives, or non-hormonal contraceptives, and who have had incorrect or discontinued use in their history, with the risk of having an unwanted pregnancy, could adopt an intrauterine device [45].

An important aspect that could influence and prolong happiness with an intrauterine device is sexual acceptability. Cramps, pain, and bleeding are the most commonly cited reasons from women for discontinuation within the first 12 months, even if they are adverse events that usually appear during the first months of use [46]. The partner could also influence the acceptability of the intrauterine device when he experiences bad sex due to the perception of the intrauterine device string during intercourse [47]. Conversely, the absence of systemic hormonal repercussions makes these intrauterine devices neutral on sexual libido compared to other hormonal methods [48]. However, authors have shown by their investigation that the QoL and sexual function improve after LNG-IUS placement. Moreover, the reduction in dysmenorrhea is another important reported aspect [44,49].

Both frequency of sexual activity and sexual enjoyment are positively related to satisfaction with a contraceptive method, as is demonstrated in other studies [50,51]. High standards of satisfaction [50] and a better QoL [52,53] have been described in IUD users who previously had unwanted pregnancies using a SARC. Furthermore, authors observed that women with sexual dysfunction experience significant improvement in sexual desire, arousal, orgasm, and overall sexual function while using LNG-IUS. Moreover, a cross-sectional investigation observed that sexual symptoms in women using LNG-IUS were the same [54] or higher than women who adopted a copper intrauterine device (IUD) [55]. 

These data are also supported by other recent studies in which healthy women who were using IUD for long-term contraception were enrolled. Among them, half used LNG-IUSs as the study group and the other half used copper IUDs. There was no significant difference in the individual score and the total scores of questionnaires used to investigate sexual function between the groups [50,56]. At first, intrauterine devices were not thought to be proper for adolescents until observational studies not only showed their contraceptive safety but also their positive role in sexuality. Therefore, new, smaller devices have encouraged doctors to suggest, and adolescents to use, intrauterine contraception. Finally, intrauterine devices do not appear to have negative interference during sexual experience, due to the absence of systemic steroid effects and the fact that they maintain the natural expression of sexuality and enjoyment during sexual intercourse [57].

### 3.5. Progestin Contraceptive Implant and Sexuality

Progestin-only contraceptive implants (POI) are subdermal devices composed of a total of 68 mg of etonogestrel (ENG), which is liberated daily at low doses of 25–70 µg on the subdermal tissue of the arm. It has the advantage of being discreet and easy to use and is classified as a LARC [58]. Some studies showed an improvement in QoL and no negative effects on libido and sexual function [54,59]. Relevant benefits to sexual function were also observed, consisting of increase in sexual pleasure, personal initiative, orgasm experience, and satisfaction; authors argued that these benefits were due to decreased anxiety and discomfort [60]. In comparative studies, greater sexual interest was observed in users of RNG implants than in users of oral contraceptives [61].

## 4. Conclusions

Today, the topic “sexuality and contraception” is a much debated field of study. It is not easy to correlate the sexual benefits or discomforts with hormonal contraception considering only the objective/biological side effects. Psychological influences can intervene before, during, and after the use of the contraceptive, hormonal or not, promoting benefits or producing no changes in sexual function [14].

During the counseling to prescribe a contraceptive, the health care practitioner (HCP) should investigate the woman’s quality of sexual life, so as to work out the method that could maintain or improve her sexuality together. At the follow-up, shared with the user in a timely manner, it will be necessary to reassess the impact of the adopted method on sexuality, and, if disturbing effects on sexuality are highlighted, opt for another contraceptive. Again, the choice of a new method must take into account the woman’s propensity and its impact on sexual function. Not infrequently, women may manifest anxiety when they experience their sexuality disturbed by the hormonal contraceptive. A review of a temporal association between the baseline of female sexual dysfunction and the introduction of contraception is warranted, as is an assessment of the bio-psycho-social exemplar of other implicit contributing elements. Attributing a sexual dysfunction to the use of a contraceptive is to be avoided at all costs. The contraceptive could also cause a sexological disorder. Researching the correct dysfunctional causes often involves an interdisciplinary approach. In fact, pain during vaginal intercourse, marital conflict, medical surgical comorbidities, or a history of sexual abuse may emerge during contraceptive counseling. A specialist consultation must be requested for each of them. 

For all these reasons, developments in oral contraception are continuing. One hormonal contraceptive cannot be the solution to all problems. In our experience, COCs containing E2 are an innovation that avoids CHC discontinuation in women suffering from acquired hypoactive sexual desire disorder during CHC intake with hypoandrogenic effects [21].

Consequently, the concept that all women are different and variably sensitive to COC steroids has to be emphasized; in fact, we are going into a historical period in which the principal concept is to advise a COC tailored to particular women. Therefore, the first step in prescribing a COC is to understand the needs of the subject and to explore her current sexual health. 

Beyond the changes in sexual function secondary to the effects of hormonal contraception, there are gray areas regarding the qualitative changes in sexual pleasure during contraceptive use. Some authors support the hypothesis that a woman’s contraceptive choice plays a role in the contraceptive effects on sexual experience and pleasure [62]. There are women who experience discomfort in being aware that they are using a contraceptive method during sex or feeling the intravaginal ring during intercourse [63].

Others consider these perceptions to favor a positive effect on the sexuality of the couple as a whole [34].

These behaviors can be generalized to all contraceptive users, nominally LARCs and SARCs. In fact, users are able to customize the use of the method while maintaining its effectiveness and to adapt it to their sexuality [64].

Sexual function in reproductive-age women can depend on the influence of sex hormones as well as on psychological, sociocultural, and relationship influences. Studies on the sexual dysfunction in women using hormonal contraceptives are single-center studies, and there are no prospective studies. Large cross-sectional and longitudinal studies have not identified a correlation between hormonal contraceptives and sexual function in women. Steroids may have a modifying effect on sexual function, but social influences are just as important [65].

Even if healthcare professionals are adequately trained to treat endocrine and gynecological disorders with hormonal contraceptives, such as chronic pelvic pain, dysmenorrhea, or hyperandrogenism, there may be evident gaps in addressing their patients’ sexual distress or sex disorder. To overcome these gaps, it is necessary to train healthcare professionals in sexual medicine and to promote better sexual well-being, including in the contraceptive field.

## Data Availability

No new data were created or analyzed in this study. Data sharing is not applicable to this article.
